# Associations between physical function and device-based measures of physical activity and sedentary behavior patterns in older adults: moving beyond moderate-to-vigorous intensity physical activity

**DOI:** 10.1186/s12877-021-02163-4

**Published:** 2021-03-31

**Authors:** Rod L. Walker, Mikael Anne Greenwood-Hickman, John Bellettiere, Andrea Z. LaCroix, David Wing, Michael Higgins, KatieRose Richmire, Eric B. Larson, Paul K. Crane, Dori E. Rosenberg

**Affiliations:** 1 Kaiser Permanente Washington Health Research Institute, 1730 Minor Ave, Suite 1600, Seattle, WA 98101 USA; 2 University of California, San Diego, 9500 Gilman Dr, La Jolla, CA 92093 USA; 3 University of Washington, 1410 NE Campus Parkway, Seattle, WA 98195 USA

**Keywords:** Physical activity, Sedentary behavior, Sedentary activity, Physical function, Physical performance, Accelerometer, Inclinometer

## Abstract

**Background:**

Research supports that moderate-to-vigorous intensity physical activity (MVPA) is key to prolonged health and function. Among older adults, substantial changes to MVPA may be infeasible, thus a growing literature suggests a shift in focus to whole-day activity patterns.

**Methods:**

With data from 795 older adults aged 65–100 in the Adult Changes in Thought Activity Monitoring study, we used linear regression to estimate associations between ActiGraph and activPAL measured activity patterns – including light intensity physical activity, steps, standing, and sedentary behaviors – and physical function as measured by a short Performance-based Physical Function (sPPF) score (range 0–12), a composite score based on three standardized physical performance tasks: gait speed, timed chair stands, and grip strength. We examined whether relationships persisted when controlling for MVPA or differed across age, gender, or quartiles of MVPA.

**Results:**

In models unadjusted for MVPA, a 1-standard deviation (SD) increment of daily sitting (1.9 h more), mean sitting bout duration (8 min longer average), or time spent in sedentary activity (1.6 h more) was associated with ~ 0.3–0.4 points lower mean sPPF score (all *p* < 0.05). A 1-SD increment in daily steps (~ 3500 more steps) was associated with ~ 0.5 points higher mean sPPF score (95% CI: 0.22 to 0.73). MVPA adjustment attenuated all relationships. The association between physical function and steps was strongest among adults aged 75+; associations of worse function with greater sedentary behavior were more pronounced in participants with the lowest levels of MVPA.

**Conclusions:**

We found associations between function and activity metrics other than MVPA in key subgroups, findings that support research on broader activity patterns and may offer ideas regarding practical intervention opportunities for improving function in older adults.

**Supplementary Information:**

The online version contains supplementary material available at 10.1186/s12877-021-02163-4.

## Background

Maintaining physical function throughout the aging process is important to the preservation of independence, the capacity to engage in physical and social activities, and quality of life [1]. The current literature examining physical activity, sedentary behavior, and health in older adults suggests that higher levels of moderate-to-vigorous intensity physical activity (MVPA) are key to prolonged health and function [2]. However, among older adults, for whom physical function typically declines with age [3, 4], increasing MVPA may not be feasible due to existing limitations in functional exercise capacity [5, 6]. Consequently, public health messages and interventions centered on boosting MVPA in this population may not be effective, optimal, or feasible [5, 7].

A growing literature suggests a focus shift to whole-day activity patterns. Emphasizing more standing [8] or light intensity physical activity [9] and reduced or broken up sedentary behavior [10] throughout the day could bring benefits of increased activity to a wider portion of the population, even without increases in MVPA [11]. Sedentary behavior, which refers to activities performed in a sitting or lying position at low energy expenditures (e.g. sitting watching television or working on the computer) [12–14], may be especially relevant for older adults as they spend, on average, over 9 h per day in sedentary activities [15–20]. Sedentary time has been associated with conditions linked to functional and physical decline, including cardiovascular disease, diabetes, and related factors (measures of insulin, glucose, and blood pressure) [21–23], and evidence is growing that links total sedentary time and patterns of sedentary time to physical function [24–27].

When shifting the paradigm of older adult activity to include whole-day activity patterns, incorporating simpler, more intuitive metrics of physical activity, like step count, will also be important. Some studies have observed associations between steps and self-reported physical function [28, 29], and others have found higher step counts to be associated with better performance on a timed-walk task [29, 30]. Steps have also been demonstrated to be inversely associated with mortality and other negative outcomes [31] and may prove to be more actionable to changing behavior.

The goal of the current study was to assess in older adults whether potential associations between objectively measured physical function and device-based measures of whole-day activity patterns – including light intensity physical activity, steps, standing time, and several sedentary behavior pattern metrics – persist beyond MVPA, and whether associations are differentiated across age, gender, or activity levels. Our overarching goal is to contribute to a more holistic understanding of physical activity and sedentary behavior relationships with functional health of older adults.

## Methods

### Setting

This manuscript used data from the Adult Changes in Thought Activity Monitoring (ACT-AM) study, a sub-study embedded within the parent ACT cohort. ACT is an on-going longitudinal cohort study that enrolls adults aged 65+ without dementia randomly selected from membership panels of Kaiser Permanente Washington (KPWA), an integrated health care delivery system in Washington state, and follows them biennially to assess changes in cognitive function and other health characteristics [32]. ACT-AM, which started in 2016, invited current ACT participants to wear at least one of two research-grade accelerometers to measure physical activity and sedentary behavior [33]. Participants who were wheelchair bound, living in a nursing home, or receiving hospice or care for a critical illness were not eligible for ACT-AM. Study procedures were approved by the KPWA institutional review board, and participants provided written informed consent.

### Activity monitoring

The accelerometers employed in ACT-AM were the waist-worn ActiGraph wGT3X+ (ActiGraph LLC, Pensacola, FL, USA), which measures sedentary, light, moderate, and vigorous intensity activity, and the thigh-worn activPAL micro (PAL Technologies, Glasgow, Scotland, UK), which measures sitting time, standing time, steps, and mean duration of sitting bouts based on body posture rather than movement. ActiGraph wGT3X+ is well-validated for measuring physical activity [34–36] and activPAL is well-validated for measuring sitting time and distinguishing among sitting patterns [37–40]; both devices have been used in many studies with older adults [41–46]. Full details of the ACT-AM device-wear protocol and data processing are available in the design paper [33]. Briefly, participants were asked to wear one or both accelerometers for a period of 7 days and to keep a log of in-bed and out-of-bed times and any periods of device-removal. An adherent wear-day was defined as having at least 10 h of awake wear time. ActiGraph data for activity intensity were processed using calibrated cut-points developed specifically for older adults based on a Women’s Health Initiative laboratory study (OPACH) [35], with vector magnitude per 15-s epoch cut-points of: < 18 for sedentary activity, 19–518 for light intensity physical activity, and > 518 for MVPA. Processing of activPAL data used proprietary PAL Technologies software and programs developed in R [47].

### Physical function

Each participant’s physical function was assessed by three in-person standardized physical performance tasks: gait speed as measured by the average of two 10-ft timed walks; chair stand time (time needed to move from a seated position in a chair to a standing position, repeated five times); and grip strength as measured by handheld dynamometer (average of three attempts in the dominant hand). As in prior ACT research, each task was scored from 0 to 4 points based on cut-points determined by sex-specific quartiles with a score of 0 indicating inability to complete the task (see eMethods, Supplemental Material); scores on each task were then summed to construct a short Performance-based Physical Function (sPPF) score ranging from 0 to 12, with higher scores indicating better physical function [48, 49]. ACT’s original Performance-based Physical Function (PPF) test was a composite of these three tasks but had also included a balance task that ACT has since discontinued, thus making it unavailable for the current study. The original ACT PPF had a Cronbach α coefficient of 0.74 and correlated with level of difficulty performing activities of daily living [48].

### Covariates

At the ACT study visit, participants provided self-reported information on demographics (age, gender, race/ethnicity, and education), exercise, difficulties with activities of daily living, and self-rated health. Body mass index (BMI) was measured from participant height and weight, depressive symptoms from questionnaire using the Center for Epidemiologic Studies Depression Scale (CES-D) [50], and cognitive function from the Cognitive Abilities Screening Instrument (CASI) [51]. Comorbidity was assessed using the Charlson Comorbidity Index [52] computed from KPWA electronic health records in the year prior to the ACT visit; diagnoses of osteoarthritis from that same year period were also recorded.

### Selection Bias

For our study we limited analyses to ACT-AM participants who had been enrolled in KPWA for at least 1 year preceding ACT-AM enrollment, whose cognitive screening assessment (e.g., CASI) did not recommend additional diagnostic evaluation for dementia, and who provided data from the sPPF and at least 4 adherent wear-days from both the ActiGraph and the activPAL devices. To account for potential selection bias due to factors related to device-wear consent and ability to wear devices and undergo performance tasks, we estimated a logistic regression model for the binary outcome of study inclusion as a function of demographic, behavioral, functional, and health characteristics (see *Covariates*), as well as whether the person’s ACT study visit was administered in the clinic or at home. Estimation of this model used the broader ACT sample initially eligible for ACT-AM who met our study’s KPWA enrollment and cognitive screening criteria. We used predictions from this model to construct inverse probability weights [53, 54] that were incorporated in all analyses described below.

### Statistical analysis

We estimated cross-sectional associations between physical function and each of the device-based activity pattern metrics using linear regression. In primary analyses, we treated physical function as measured by sPPF as the dependent variable, and the pattern metrics served as exposures of interest (continuous). We fit a separate model for each exposure including: sitting time; standing time; mean sitting bout duration; steps; and time spent in sedentary activity, light intensity physical activity, and MVPA. In secondary analyses, we estimated associations with each of the individual physical function task outcomes separately (gait speed, chair time, grip strength), limiting to participants able to complete each of the respective tasks. In both primary and secondary analyses, we adjusted models for awake wear time using the residual method [55] and initially included covariate adjustment for age (continuous), gender (female vs. male), race/ethnicity (non-Hispanic white vs. other), education (post-secondary education vs. high school or less), BMI (using natural cubic splines [56]), depressive symptoms (CES-D score 10+ vs. < 10), osteoarthritis (yes vs. no), and Charlson Comorbidity Index (continuous). We then additionally adjusted for MVPA to assess whether any potential associations between physical function and the other pattern metrics remained when controlling for time spent at higher intensity activities. All models incorporated inverse probability weights to account for selection into the analytic sample (described earlier), and model parameters were estimated using weighted generalized estimating equations with standard errors estimated via the sandwich estimator [57].

As additional sensitivity analyses, we re-estimated the above associations allowing for non-linear relationships between physical function and the activity pattern metrics. We did this by representing the exposures using splines rather than single continuous terms. Additionally, we performed three sets of pre-specified subgroup analyses, estimating the associations between sPPF and the activity pattern metrics in groups defined by age (65–74, 75–84, 85+), gender, or quartiles of MVPA. Models used to estimate these associations incorporated interaction terms between the activity pattern metric and subgroup indicators and included the same selection weighting and adjustment variables (including MVPA) as used in primary analyses. All analyses were conducted using R, version 3.5.3 [47].

## Results

A total of 813 ACT-AM participants met study inclusion criteria. We excluded 18 (2%) people due to missing covariate information (3 missing race/ethnicity, 7 missing BMI, and 8 missing CES-D score), resulting in a final analytic sample of 795 participants. Characteristics of this sample, overall and by age groups, are provided in Table 1. More than half of participants were aged 75 or older, and over 90% had education beyond high school. A majority were overweight or obese, and approximately 40% had one or more comorbidities (as measured by Charlson). Table 1 also includes distribution summary statistics for the sPPF and the device-based activity pattern metrics of interest after weighting to account for selection into the analytic sample (see eFigure 1, Supplemental Material which displays histograms of the unweighted distributions). On average, older adults sat for 10 h per day with a mean sitting bout duration of 16 min, accumulated approximately 6500 steps, spent more than 4 h doing LPA, and about an hour doing MVPA; the mean sPPF score was 8.3 (standard deviation 2.9).

**Table 1:** Characteristics of included ACT-AM participants and summary statistics for the sPPF and activity pattern metrics

	All ages		Age 65–74		Age 75–84		Age 85+	
	N	%	N	%	N	%	N	%
Total	795		355		330		110	
Age*, mean (SD)*	76.7 (6.8)		70.8 (2.3)		78.9 (2.9)		89.0 (3.6)	
Female	434	55	188	53	181	55	65	59
Non-Hispanic white	720	91	329	93	293	89	98	89
Post-secondary education	730	92	339	95	304	92	87	79
Body mass index*, mean (SD)*	26.8 (4.7)		27.1 (5.1)		26.8 (4.4)		26.3 (4.3)	
< 25	307	39	141	40	124	38	42	38
25- < 30	317	40	134	38	138	42	45	41
30+	171	22	80	23	68	21	23	21
Osteoarthritis	172	22	72	20	74	22	26	24
Depressive symptoms (CES-D score ≥ 10)	68	9	26	7	31	9	11	10
Charlson comorbidity index								
0	470	59	246	69	185	56	39	35
1	129	16	59	17	52	16	18	16
2	99	12	31	9	47	14	21	19
3+	97	12	19	5	46	14	32	29
Regular exercise (15 min ≥3 times/week)	641	81	299	84	265	80	77	70
Difficulty with ≥1 activities of daily living	144	18	39	11	73	22	32	29
Fair/Poor self-rated health	53	7	12	3	23	7	18	16
sPPF^a^, *mean (SD)*	8.3 (2.9)		9.4 (2.3)		8.4 (2.8)		6.5 (3.0)	
sPPF^a^, *median (quartile 1, quartile 3)*	9 (7, 10)		10 (8, 11)		9 (7, 10)		7 (4, 9)	
Activity pattern metrics^a,b^*, mean (SD)*								
activPAL								
MBD, minutes	16.0 (8.0)		14.9 (7.8)		16.4 (7.8)		17.1 (8.6)	
Sitting hours	10.1 (1.9)		9.8 (1.9)		10.0 (2.0)		10.7 (1.8)	
Standing hours	4.0 (1.6)		4.1 (1.5)		4.1 (1.7)		3.8 (1.7)	
Step count	6453 (3474)		7803 (3774)		6259 (3103)		4460 (2398)	
ActiGraph								
SA hours	9.6 (1.6)		9.2 (1.6)		9.6 (1.6)		10.4 (1.2)	
LPA hours	4.6 (1.2)		4.6 (1.2)		4.7 (1.3)		4.3 (1.1)	
MVPA hours	1.0 (0.7)		1.4 (0.7)		1.0 (0.7)		0.5 (0.4)	

*Note. ACT-AM* Adult Changes in Thought - Activity Monitoring sub-study, *SD* Standard deviation, *CES-D* Center for Epidemiologic Studies Depression Scale, *sPPF* Short Performance-based Physical Function score, *MBD* Mean sitting bout duration, *SA* Sedentary activity, *LPA* Light intensity physical activity, *MVPA* Moderate-to-vigorous intensity physical activity

^a^Summary statistics for the sPPF and the device-based activity pattern metrics incorporate weighting to account for selection into the analytic sample

^b^Average daily measures from ActiGraph and activPAL are adjusted for awake wear time

Table 2 provides the estimated associations between physical function and each of the device-based activity pattern metrics, with and without adjustment for MVPA. Estimates and 95% confidence intervals (CI) correspond to differences in mean sPPF score (or individual task measures) associated with a 1-standard deviation (SD) difference in the given activity pattern metric. For example, in models not adjusted for MVPA, estimates for the sedentary pattern metrics showed that a 1-SD increment in amount of daily sitting time (1.9 h more) or mean sitting bout duration (8 min longer average) from activPAL or a 1-SD increment in time spent in sedentary activity (1.6 h more) from ActiGraph was associated with approximately 0.3 to 0.4 points lower mean sPPF score. Specific estimates were − 0.29 (95% CI: − 0.54 to − 0.04) for sitting, − 0.37 (95% CI: − 0.72 to − 0.01) for mean sitting bout duration, and − 0.38 (95% CI: − 0.62 to − 0.14) for sedentary activity. Daily total sitting time and mean sitting bout duration were not significantly associated with any of the individual function test components (only the composite sPPF score), but sedentary activity did show an additional association with gait speed specifically. Once we adjusted for MVPA, however, all associations between the sedentary pattern metrics and physical function were attenuated and no longer statistically significant. In regard to physical activity metrics, there were no significant associations between physical function and light intensity physical activity, but results for total steps and MVPA showed that a 1-SD increment in daily steps (~ 3500 more) or MVPA (~ 45 min more) was associated with approximately 0.5 points higher mean sPPF score. Specific estimates were 0.47 (95% CI: 0.22 to 0.73) for steps and 0.48 (95% CI: 0.24 to 0.72) for MVPA. These latter metrics were also associated with individual physical function components, including gait speed and chair stands, but not with grip strength. Adjusting for MVPA attenuated the association between steps and physical function.

**Table 2:** Estimated associations^a^ between the performance-based physical function measures and the device-based activity pattern metrics

		sPPF, *N* = 795 Mean (SD) = 8.3 (2.9)		Gait speed (m/s), *N* = 785 Mean (SD) = 0.9 (0.2)		Chair time (s), *N* = 720 Mean (SD) = 12.3 (4.0)		Grip strength (kg), *N* = 739 Mean (SD) = 25.1 (9.6)	
		Model 1^b^	Model 2^c^	Model 1^b^	Model 2^c^	Model 1^b^	Model 2^c^	Model 1^b^	Model 2^c^
Average daily:	Mean (SD)	Estimate (95% CI)	Estimate (95% CI)	Estimate (95% CI)	Estimate (95% CI)	Estimate (95% CI)	Estimate (95% CI)	Estimate (95% CI)	Estimate (95% CI)
**MBD, minutes**	16.0 (8.0)	−0.37* (−0.72, −0.01)	−0.28 (−0.66, 0.09)	−0.01 (−0.03, 0.00)	−0.00 (− 0.01, 0.01)	0.42 (− 0.01, 0.85)	0.32 (− 0.14, 0.77)	0.22 (− 0.32, 0.76)	0.35 (− 0.23, 0.93)
**Sitting hours**	10.1 (1.9)	− 0.29* (− 0.54, − 0.04)	− 0.18 (− 0.45, 0.09)	−0.01 (− 0.02, 0.01)	0.00 (− 0.01, 0.02)	0.32 (− 0.05, 0.69)	0.20 (− 0.21, 0.61)	−0.24 (− 0.76, 0.29)	−0.12 (− 0.66, 0.43)
**Standing hours**	4.0 (1.6)	0.18 (−0.06, 0.42)	0.14 (− 0.10, 0.38)	0.00 (− 0.02, 0.02)	− 0.00 (− 0.02, 0.01)	−0.13 (− 0.50, 0.23)	−0.10 (− 0.47, 0.28)	0.15 (− 0.36, 0.66)	0.11 (− 0.39, 0.62)
**Step count**	6453 (3474)	0.47* (0.22, 0.73)	0.28 (− 0.06, 0.61)	0.03* (0.01, 0.04)	−0.01 (− 0.03, 0.02)	−0.74* (−1.06, − 0.41)	−0.69* (−1.17, − 0.21)	0.27 (− 0.30, 0.85)	−0.09 (− 0.88, 0.70)
**SA hours**	9.6 (1.6)	− 0.38* (− 0.62, − 0.14)	− 0.15 (− 0.48, 0.18)	−0.02* (− 0.04, − 0.01)	0.00 (− 0.02, 0.02)	0.33 (− 0.04, 0.71)	0.03 (−0.46, 0.52)	− 0.12 (− 0.67, 0.44)	0.27 (− 0.41, 0.95)
**LPA hours**	4.6 (1.2)	0.19 (− 0.06, 0.43)	0.11 (− 0.14, 0.37)	0.00 (− 0.01, 0.02)	−0.00 (− 0.02, 0.01)	−0.11 (− 0.49, 0.26)	−0.03 (− 0.40, 0.35)	−0.12 (− 0.64, 0.40)	−0.21 (− 0.74, 0.32)
**MVPA hours**	1.0 (0.7)	0.48* (0.24, 0.72)	NA	0.05* (0.03, 0.06)	NA	−0.52* (− 0.86, − 0.17)	NA	0.49 (− 0.09, 1.07)	NA

*Note. sPPF* Short Performance-based Physical Function score, *MBD* Mean sitting bout duration, *SA* Sedentary activity, *LPA* Light intensity physical activity, *MVPA* Moderate-to-vigorous intensity physical activity, *SD* Standard deviation, *CI* Confidence interval

^a^Estimates correspond to differences in means of the performance-based physical function measure associated with a 1-SD difference in the device-based activity pattern metric. All estimates incorporate weighting to account for selection into the analytic sample

^b^Model 1 includes adjustment for awake wear time, age, gender, race/ethnicity, education, body mass index, osteoarthritis, depressive symptoms, and Charlson comorbidity index

^c^Model 2 is the same as Model 1 but includes additional adjustment for MVPA

*p < 0.05

Results from sensitivity analyses using splines to allow for non-linear relationships between physical function and the activity pattern metrics are presented in Table 3. This table summarizes these relationships by providing model estimates of adjusted means of physical function measures at the 25th, 50th, and 75th percentiles of the distributions of the activity pattern metrics. Adjustment variables were the same as in primary analyses and included adjustment for MVPA. While these sensitivity analyses did suggest evidence of some non-linear relationships between most of the pattern metrics and sPPF (including grip strength, specifically), the general trends observed were similar with those from primary analyses, particularly when comparing differences in mean scores between the 25th and 75th percentiles. To help illustrate this point, eFigure 2, Supplemental Material provides plots of the estimated linear vs. spline models to show the general conclusions are mostly unchanged by this added flexibility.

**Table 3:** Adjusted means of physical function measures at select percentiles of the activity pattern metric distributions

Average daily:	sPPF Adj. Mean (95% CI)	Gait speed (m/s) Adj. Mean (95% CI)	Chair time (s) Adj. Mean (95% CI)	Grip Strength (kg) Adj. Mean (95% CI)
**MBD, minutes**	*			
25th %ile: 11.4 min	8.5 (8.2, 8.8)	0.89 (0.87, 0.92)	12.1 (11.5, 12.6)	24.8 (23.9, 25.6)
50th %ile: 14.5 min	8.7 (8.4, 9.0)	0.89 (0.87, 0.91)	12.4 (12.0, 12.9)	25.5 (24.7, 26.2)
75th %ile: 18.4 min	8.3 (8.0, 8.6)	0.88 (0.86, 0.91)	12.8 (12.3, 13.2)	25.4 (24.7, 26.1)
**Sitting hours**	*			*
25th %ile: 8.9 h	8.5 (8.2, 8.8)	0.88 (0.86, 0.90)	12.3 (11.8, 12.8)	25.9 (25.1, 26.6)
50th %ile: 10.2 h	8.7 (8.4, 9.0)	0.89 (0.86, 0.91)	12.4 (11.9, 12.9)	25.3 (24.5, 26.2)
75th %ile: 11.3 h	8.4 (8.1, 8.8)	0.90 (0.87, 0.92)	12.7 (12.1, 13.3)	24.7 (24.0, 25.5)
**Standing hours**				*
25th %ile: 3.0 h	8.4 (8.1, 8.8)	0.90 (0.87, 0.92)	12.6 (12.1, 13.2)	24.6 (23.8, 25.4)
50th %ile: 3.9 h	8.6 (8.3, 9.0)	0.88 (0.86, 0.91)	12.5 (11.9, 13.0)	25.4 (24.6, 26.2)
75th %ile: 4.9 h	8.5 (8.2, 8.8)	0.88 (0.86, 0.90)	12.4 (12.0, 12.9)	25.8 (25.1, 26.6)
**Step count**	*	*		*
25th %ile: 3992 steps	8.1 (7.7, 8.6)	0.89 (0.86, 0.92)	13.1 (12.4, 13.8)	25.1 (24.2, 26.1)
50th %ile: 6031 steps	8.7 (8.4, 9.0)	0.90 (0.88, 0.92)	12.4 (11.9, 12.8)	25.0 (24.2, 25.8)
75th %ile: 8431 steps	8.9 (8.6, 9.1)	0.90 (0.88, 0.92)	11.8 (11.3, 12.3)	25.4 (24.6, 26.1)
**SA hours**	*			*
25th %ile: 8.6 h	8.6 (8.3, 8.9)	0.89 (0.87, 0.92)	12.4 (11.8, 13.0)	25.3 (24.5, 26.2)
50th %ile: 9.6 h	8.7 (8.3, 9.0)	0.90 (0.87, 0.93)	12.3 (11.8, 12.8)	25.5 (24.7, 26.4)
75th %ile: 10.7 h	8.2 (7.8, 8.6)	0.89 (0.86, 0.92)	12.7 (12.0, 13.4)	25.2 (24.2, 26.1)
**LPA hours**				*
25th %ile: 3.7 h	8.2 (7.8, 8.5)	0.88 (0.86, 0.91)	12.6 (12.0, 13.1)	24.7 (24.0, 25.5)
50th %ile: 4.4 h	8.6 (8.3, 9.0)	0.89 (0.87, 0.92)	12.5 (12.0, 13.0)	25.9 (25.0, 26.8)
75th %ile: 5.3 h	8.5 (8.2, 8.8)	0.90 (0.88, 0.92)	12.5 (11.9, 13.0)	25.4 (24.6, 26.1)
**MVPA hours**	*	*		*
25th %ile: 0.5 h	7.8 (7.4, 8.2)	0.86 (0.83, 0.88)	13.1 (12.4, 13.7)	24.8 (23.9, 25.8)
50th %ile: 0.9 h	8.6 (8.3, 8.9)	0.89 (0.87, 0.92)	12.6 (12.1, 13.4)	25.1 (24.3, 25.9)
75th %ile: 1.5 h	9.0 (8.7, 9.3)	0.93 (0.91, 0.95)	11.9 (11.5, 12.4)	25.8 (25.1, 26.5)

*Note. sPPF* Short Performance-based Physical Function score, *MBD* Mean sitting bout duration, *SA* Sedentary activity, *LPA* Light intensity physical activity, *MVPA* Moderate-to-vigorous intensity physical activity; *Adj*. Adjusted, *CI* Confidence interval

*p < 0.05 for comparison of spline vs. linear model

^a^Estimates are based on regression models that use natural cubic splines to allow for potential non-linear associations and that include adjustment for awake wear time, age, gender, race/ethnicity, education, body mass index, osteoarthritis, depressive symptoms, Charlson comorbidity index, and MVPA. All estimates incorporate weighting to account for selection into the analytic sample

Table 4 displays results from subgroup analyses, with estimates corresponding to differences in mean sPPF score associated with a 1-SD difference in the activity pattern metric. Associations of better function with higher daily standing time, step count, light intensity physical activity, and MVPA appeared to be of greater magnitude for older age groups. For example, among the 65–74, 75–84, and 85+ age groups, the estimated effect sizes for step count were − 0.01 (95% CI: − 0.33 to 0.31), 0.41 (95% CI: 0.06 to 0.75), and 0.93 (95% CI: 0.40 to 1.45), respectively (p-interaction< 0.001). We did not observe significant differences in associations by gender for any of the activity pattern metrics except mean sitting bout duration (p-interaction = 0.027), with greater duration of this latter metric appearing to be associated with worse physical function in women (− 0.65; 95% CI: − 1.22 to − 0.08) but not men (0.06; 95% CI: − 0.25 to 0.37). Estimates of associations between physical function and the sedentary-related activity pattern metrics (sitting time, mean sitting bout duration, and time in sedentary activity) differed by quartile of MVPA (p-interaction< 0.002 for each metric). Specifically, associations of worse function with higher levels of sedentary-related metrics tended to be limited to participants with the lowest levels of MVPA.

**Table 4:** Estimated associations^a^ between the sPPF and activity pattern metrics in age, gender, and MVPA subgroups

	Age			Gender		Quartiles of MVPA (hours)			
	65–74	75–84	85+	Women	Men	< 0.52	[0.52, 0.95)	[0.95, 1.45)	1.45+
Average daily:	Estimate (95% CI)	Estimate (95% CI)	Estimate (95% CI)	Estimate (95% CI)	Estimate (95% CI)	Estimate (95% CI)	Estimate (95% CI)	Estimate (95% CI)	Estimate (95% CI)
**MBD, minutes**	−0.04 (−0.32, 0.24)	−0.26 (−0.64, 0.12)	− 0.69 (−1.43, 0.04)	− 0.65* (−1.22, − 0.08)	**0.06 (− 0.25, 0.37)**	− 0.43* (− 0.86, 0.00)	**−0.33 (− 0.82, 0.16)**	**0.08 (− 0.25, 0.40)**	**0.07 (− 0.32, 0.45)**
**Sitting hours**	−0.14 (− 0.40, 0.12)	−0.16 (− 0.44, 0.12)	−0.28 (− 0.64, 0.08)	−0.34 (− 0.70, 0.02)	0.08 (− 0.27, 0.43)	−0.34* (− 0.63, − 0.06)	**−0.20 (− 0.48, 0.08)**	**−0.03 (− 0.28, 0.22)**	**0.04 (− 0.23, 0.31)**
**Standing hours**	**−0.07 (− 0.32, 0.18)**	**0.16 (− 0.10, 0.42)**	**0.35 (0.00, 0.69)**	0.29 (− 0.04, 0.62)	−0.10 (− 0.43, 0.23)	0.07 (− 0.23, 0.37)	0.15 (− 0.15, 0.46)	0.30* (0.05, 0.56)	0.18 (− 0.08, 0.45)
**Step count**	**−0.01 (− 0.33, 0.31)**	0.41* (0.06, 0.75)	0.93* (0.40, 1.45)	0.48* (0.06, 0.90)	0.13 (−0.24, 0.50)	0.48 (− 0.21, 1.16)	0.43* (0.02, 0.84)	0.37* (0.03, 0.70)	0.06 (−0.31, 0.44)
**SA hours**	−0.11 (− 0.44, 0.21)	−0.12 (− 0.45, 0.21)	−0.19 (− 0.56, 0.18)	−0.24 (− 0.63, 0.15)	0.04 (− 0.34, 0.42)	**−0.26 (− 0.59, 0.08)**	**−0.15 (− 0.50, 0.20)**	**0.00 (− 0.33, 0.33)**	**0.07 (− 0.27, 0.40)**
**LPA hours**	**−0.05 (− 0.30, 0.20)**	**0.17 (− 0.10, 0.43)**	**0.32 (− 0.04, 0.68)**	0.27 (− 0.05, 0.60)	−0.17 (− 0.50, 0.17)	−0.07 (− 0.42, 0.28)	0.05 (− 0.24, 0.34)	0.19 (− 0.07, 0.45)	0.17 (− 0.08, 0.43)
**MVPA hours**	0.30* (0.03, 0.57)	0.63* (0.37, 0.90)	0.82* (0.21, 1.42)	0.45* (0.15, 0.74)	0.53* (0.19, 0.86)				

*Note. sPPF* Short Performance-based Physical Function score, *MBD* Mean sitting bout duration, *SA* Sedentary activity, *LPA* Light intensity physical activity, *MVPA* Moderate-to-vigorous intensity physical activity, *SD* Standard deviation, *CI* Confidence interval

*p < 0.05

^a^Estimates correspond to differences in means of the sPPF associated with a 1-SD difference in the device-based activity pattern metric (SDs same as reported in Table 2). Models included adjustment for awake wear time, age, gender, race/ethnicity, education, body mass index, osteoarthritis, depressive symptoms, Charlson comorbidity index, and MVPA, and incorporated weighting to account for selection into the analytic sample. **Bolded** results indicate a significant interaction (*p* < 0.05) by age, gender, or quartiles of MVPA, respectively

## Discussion

Using device-measured activity pattern metrics and objective evaluations of physical function on a community-dwelling sample of older adults, we found that higher levels of sedentary behaviors were associated with worse overall function, while greater daily steps and MVPA (but not light intensity physical activity) were associated with better function when looking at all age groups combined. These associations with sedentary behaviors were with an overall measure of physical function (sPPF) rather than any one physical function component. Associations with total steps and MVPA, however, were found for both the overall measure and the individual timed gait and chair stand components. Notably, all observed associations between activity pattern metrics and overall physical function in primary analyses were relatively small in magnitude, such as differences of 0.3–0.5 points on the sPPF and ≤ 0.05 m/s in gait speed for each 1-SD difference in pattern metric, and all of these associations were attenuated when controlling for level of MVPA.

Perera et al. estimated that a 0.5 point difference in the Short Performance Physical Battery (SPPB) [58], on which the sPPF is modeled, and a 0.05 m/s difference in gait speed constitute small but clinically meaningful within-person changes in older adults, with more clinically substantial changes represented by effect sizes twice that size [59]. Researchers using data from the LIFE-P study reached similar conclusions that 0.03–0.05 m/s differences in gait speed and 0.3–0.8 point differences on the SPPB represent minimally significant within-person changes [60]. Aligning with the current literature base that focuses on MVPA in older adults as a key factor associated with maintaining function with age, the strongest associations with physical function that we observed were for MVPA from ActiGraph and steps from activPAL, two measures that were highly correlated with each other in our data (r = 0.77). Magnitudes of these associations were perhaps even more pronounced in sensitivity analyses allowing for non-linear relationships (as evidenced in eFigure 2, Supplemental Material), with differences in mean sPPF of approximately 1–2 points when contrasting those at the highest and lowest levels of activity. Importantly, though, given the cross-sectional nature of these data, such associations could reflect reverse or bi-directional causation in that more intact physical function is needed for many types of MVPA. In addition, even if such associations reflected causal relationships, differences in activity of that size are likely unrealistic as a basis for potential intervention targets in older adults due to the magnitude of behavior change required (e.g., increasing daily steps from 3000/day to nearly 9000/day or increasing daily MVPA from 15 min/day to at least 90 min/day) and the existing diversity in older adults’ functional exercise capacity and willingness to routinely engage in this much activity [5, 6].

Though effect sizes of associations in our study were small, we see comparable findings in the research literature around relationships between device-assessed activity pattern metrics and physical function, though not always when considering associations with sedentary patterns controlled for MVPA. Among adults aged 60–64 in the British Cohort Study for whom sedentary time and MVPA were measured via Actiheart, a 1-SD greater time spent sedentary (2.1 h/day more) was associated with a 0.01 m/s slower mean gait speed and 0.54 kg weaker mean grip strength, while a 1-SD greater time spent doing MVPA (1.0 h/day more) was associated with a 0.02 m/s faster gait speed and 0.48 kg stronger grip strength [61]. In the Maastricht Study (~ 1900 adults aged 40–75 in the Netherlands), researchers found higher total sitting and longer mean sitting bout duration were weakly associated with lower physical function as measured by timed walk, chair rise, and grip strength tasks (they did not consider a composite measure) [62]. Yet like in our study, after adjusting for comorbidities and an activPAL derived measure of higher intensity physical activity, these associations were attenuated (though some retained statistical significance). Further, those researchers found that physical function had stronger relationships with physical activity metrics (particularly higher intensity) than with sedentary metrics, a result similar to our finding with the activPAL measure of step count and the ActiGraph derived measure of MVPA. Davis et al. analyzed data from Project OPAL (adults aged 70+) and found that higher levels of ActiGraph assessed MVPA were associated with greater SPPB scores; however, unlike our study, they observed significant associations between greater sedentary behavior and worse SPPB scores (and the individual function components) even after controlling for MVPA [25]. Other studies in older adults have also found similar associations to persist even after controlling for MVPA [27, 63].

A relatively novel component of our study was the ability to examine whether associations between activity pattern metrics and physical function differed by age, gender, or level of MVPA. The only significant difference by gender was the association of worse sPPF with longer mean sitting bout duration for women but not men. This is a novel finding, which combined with previous evidence that older women accumulate their sedentary time in notably different patterns than older men [64], may warrant future research. When examining associations by age, we found the magnitudes of association between sPPF and physical activity (standing, steps, light intensity physical activity, and MVPA) to be greater with older age. The association with total steps, in particular, was notable as it was of greater magnitude in the older groups even after controlling for MVPA. Step count is perhaps a more clearly actionable (and easily measurable) intervention target for older adults. When we stratified analyses by level of MVPA, we observed that associations between sedentary patterns and physical function were limited to participants with the lowest levels of MVPA. This latter finding aids our interpretation of the lack of association observed between sedentary metrics (sitting time, mean sitting bout duration, and time in sedentary activity) and physical function in the primary analyses that simply controlled for MVPA, as it suggests there may still be important associations between sedentary patterns and function that could be leveraged for intervention among older adults with little to no MVPA.

The interactions we found are consistent with prior literature. Analyses of adults in the AusDiab study found that the association between stepping time (including both light and MVPA stepping) and gait and mobility varied notably by age, with the strongest association observed among the oldest participants [65]. A study of community-dwelling older adults in Japan found that sedentary patterns were more strongly associated with measures of mobility function in women than in men [66]. Finally, research by Keevil et al. using the EPIC-Norfolk study investigated associations between ActiGraph-measured sedentary time and grip strength, gait speed, and timed chair stands stratified by levels of MVPA [67]. As in our study, they observed that associations were limited to participants in the lowest quartile of MVPA.

A key strength of our study was the ability to obtain objective measures of both sedentary patterns and physical activity through participants’ concurrent wear of two different monitoring devices. We also had a relatively large sample of participants who wore devices, thus allowing meaningful subgroup analyses. Further, because we had information on the broader ACT sample who did not consent to device-wear or meet other study inclusion criteria, we were able to account for selection into our sample and provide estimated associations that may be more generalizable to older adults. Still, we acknowledge portability of findings to some populations may be limited as the ACT sample is predominately non-Hispanic white and has relatively high educational attainment. The sample also appears to have high MVPA, but it is important to note we used ActiGraph cut-points specifically developed for older adults [35]. These cut-points result in notably more time being classified as MVPA than would have been classified had we used the cut-points frequently applied in other research studies of older adults, cut-points that were derived from younger populations. We consider this more appropriate for research on older adult activity but recognize it can inhibit comparisons with other studies. Other weaknesses of our study were its cross-sectional nature, preventing the disentangling of what is likely a reciprocal relationship between declining activity and declining physical function, and the inability to compute the more standard SPPB functional measure or the original full PPF using current ACT data. Access to data from a balance test would have provided us with another key measure of function, as well as made comparisons with other literature more straightforward. We have moved toward adding the balance test to the battery of information collected at future ACT visits. Also, a next step in our research will be to build on our findings and investigate how these sedentary and physical activity metrics predict future change in physical function, as we are in the process of collecting biennial follow-up data on this ACT sample.

## Conclusions

Overall, our study adds to the body of literature showing cross-sectional relationships between physical function and device-measured activity patterns, with MVPA standing out most prominently in relation to differences in function. However, crucial to moving beyond a focus on MVPA in older adults, our findings suggest potentially important associations between function and other activity metrics in key subgroups, namely step counts among the oldest adults and sedentary patterns among those with low MVPA. These findings may provide insight when thinking about practical intervention opportunities for improving function in older adults, particularly when targeting those aged 75+ or those with limited involvement in higher intensity activities due to functional capacity.

## Supplementary Information

**Additional file 1 Supplemental Material.**

## Data Availability

The datasets used and/or analyzed during the current study are available from the corresponding author on reasonable request.

## Abbreviations

MVPA: Moderate-to-vigorous intensity physical activity
ACT: Adult Changes in Thought
ACT-AM: Adult Changes in Thought - Activity Monitoring sub-study
KPWA: Kaiser Permanente Washington
OPACH: Objective Physical Activity and Cardiovascular Disease Health in Older Women Study
sPPF: Short Performance-based Physical Function
PPF: Performance-based Physical Function
BMI: Body mass index
CES-D: Center for Epidemiologic Studies Depression Scale
CASI: Cognitive Abilities Screening Instrument
SD: Standard deviation
CI: Confidence Interval
SPPB: Short Performance Physical Battery
