# Supplementary material for: Associations between physical function and device-based measures of physical activity and sedentary behavior patterns in older adults: moving beyond moderate-to-vigorous intensity physical activity
Source: BMC Geriatr. 2021 Mar 31;21:216. doi: 10.1186/s12877-021-02163-4 (PMC8011072; doi:10.1186/s12877-021-02163-4)
Supplement: Supplementary file 1 — Additional file 1 Supplemental Material. [file 12877_2021_2163_MOESM1_ESM.docx]

Associations between Physical Function and Device-based Measures of Physical Activity and Sedentary Behavior Patterns in Older Adults: Moving Beyond Moderate-to-Vigorous Intensity Physical Activity

Rod L. Walker, MS^1^; Mikael Anne Greenwood-Hickman, MPH^1^; John Bellettiere, PhD, MPH^2^; Andrea Z. LaCroix, PhD, MPH^2^; David Wing, MS^2^; Michael Higgins, MS^2^; KatieRose Richmire, BS^1^; Eric B. Larson, MD, MPH^1^; Paul K. Crane, MD, MPH^3^; Dori E. Rosenberg, PhD, MPH^1^

^1^Kaiser Permanente Washington Health Research Institute, 1730 Minor Ave, Suite 1600, Seattle, WA, 98101, USA

^2^University of California, San Diego, 9500 Gilman Dr, La Jolla, CA, 92093, USA

^3^University of Washington, 1410 NE Campus Parkway, Seattle, WA, 98195, USA

**Supplemental Material**

|  |  | **Page** |
| --- | --- | --- |
| **eMethods** | Calculation of the short Performance-based Physical Function (sPPF) score | **3** |
| **eFigure1** | Histograms of the device-based activity pattern metrics and sPPF scores among the 795 participants included in analyses | **4** |
| **eFigure2** | Linear vs. nonlinear associations between the short Performance-based Physical Function score and the device-based activity pattern metrics | **5** |

**eMethods.** Calculation of the short Performance-based Physical Function (sPPF) score.

*Physical function as measured by sPPF*

Each participant’s physical function was assessed by three in-person standardized physical performance tasks: gait speed as measured by the average of two 10-foot timed walks; chair stand time (time needed to move from a seated position in a chair to a standing position, repeated five times); and grip strength as measured by handheld dynamometer (average of three attempts in the dominant hand). As in prior ACT research, each task was scored from 0 to 4 points based on cut-points determined by sex-specific quartiles (see below). Scores on each task were then summed to construct the sPPF score ranging from 0 to 12, with higher scores indicating better physical function.

*10-foot timed walk*

The average time of the two walks was scored as:

0 = unable to do;

1 = more than 4.5 seconds for men, more than 5.0 seconds for women;

2 = 4.0 to 4.5 seconds for men, 4.0 to 5.0 seconds for women;

3 = 3.0 to 4.0 seconds for both men and women;

4 = 3.0 seconds or less for both men and women.

*Timed chair stands*

The time to complete 5 chair stands was scored as:

0 = unable to do;

1 = more than 20 seconds for men, more than 21 seconds for women;

2 = 17 to 20 seconds for men, 18 to 21 seconds for women;

3 = 11 to 17 seconds for men, 12 to 18 seconds for women;

4 = 11 seconds or less for men, 12 seconds or less for women.

*Grip strength*

The average strength of the dominant hand was scored as:

0 = unable to do;

1 = less than 25.0 kg for men, less than 15.0 kg for women;

2 = 25.0 to 30.0 kg for men, 15.0 to 20.0 kg for women;

3 = 30.0 to 40.0 kg for men, 20.0 to 25.0 kg for women;

4 = 40.0 kg or more for men, 25.0 kg or more for women.

**eFigure 1.** Histograms of the device-based activity pattern metrics and sPPF scores among the 795 participants included in analyses.

**
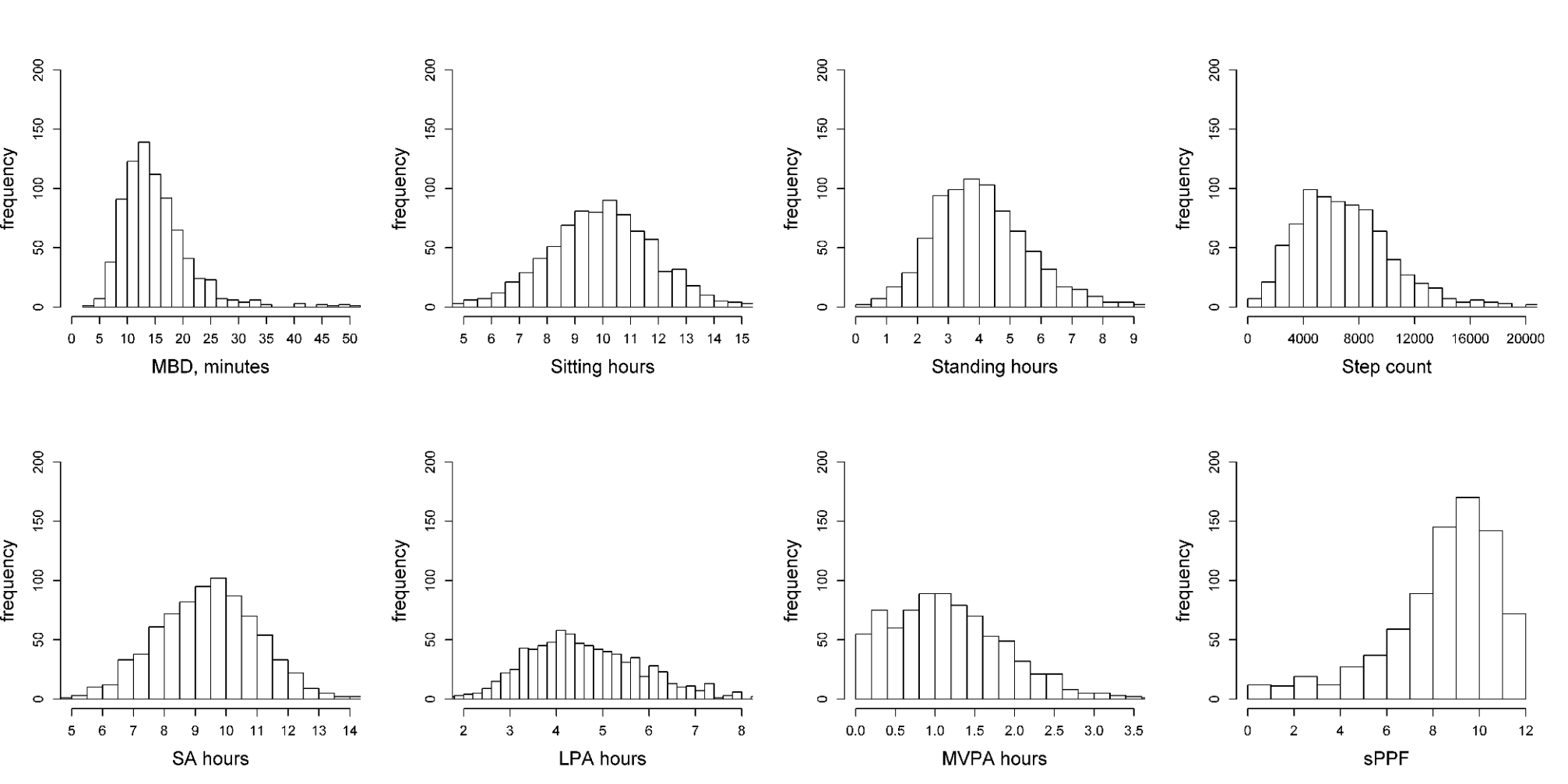
**

*Note.* sPPF = short Performance-based Physical Function score; MBD = mean sitting bout duration; SA = sedentary activity; LPA = light intensity physical activity; MVPA = moderate-to-vigorous intensity physical activity.

activPAL measures include MBD, sitting, standing, and steps. ActiGraph measures include SA, LPA, and MVPA

**eFigure 2.** Linear vs. nonlinear associations between the sPPF score and the device-based activity pattern metrics.

**
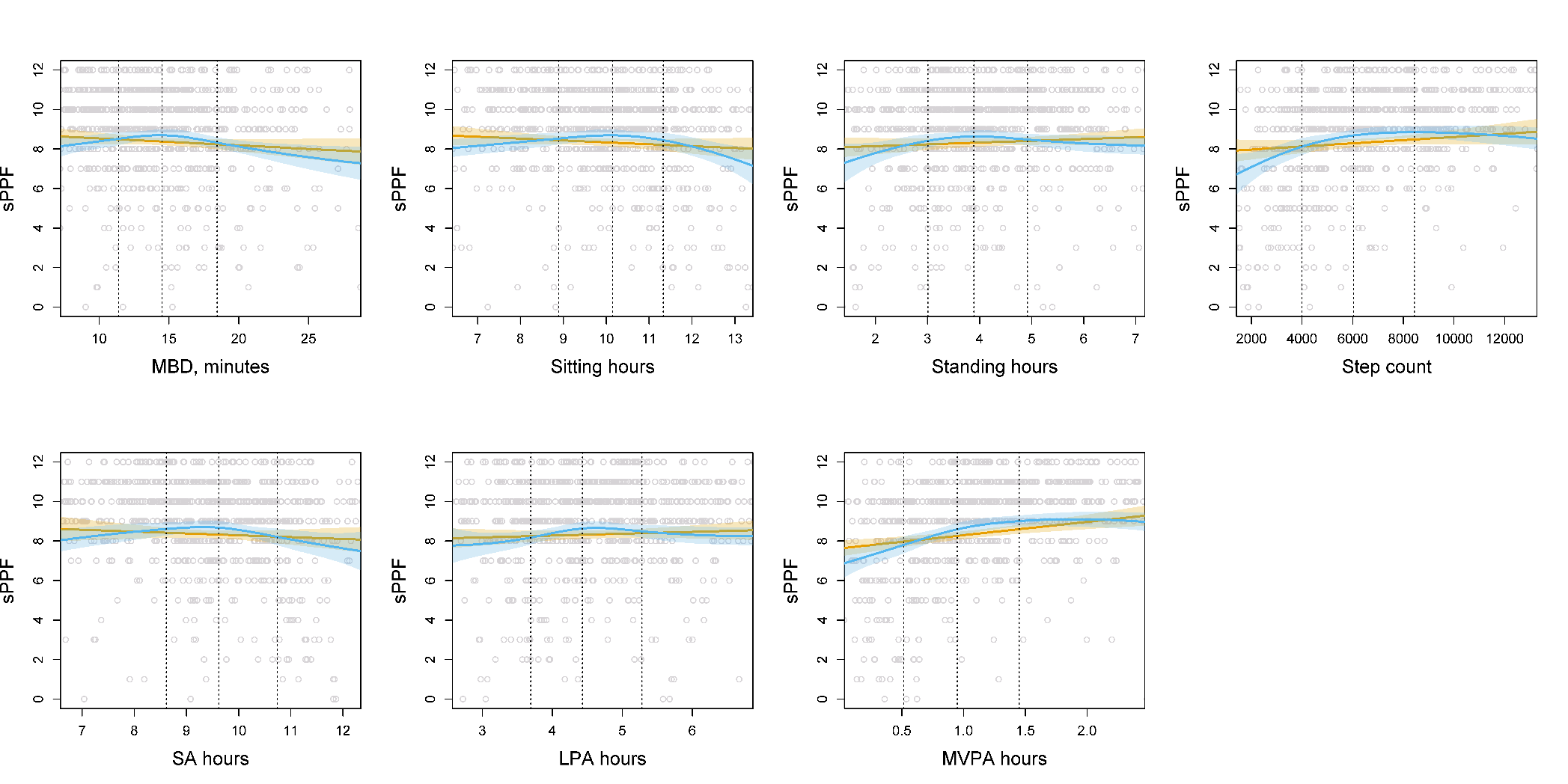
**

*Note.* sPPF = short Performance-based Physical Function score; MBD = mean sitting bout duration; SA = sedentary activity; LPA = light intensity physical activity; MVPA = moderate-to-vigorous intensity physical activity.

activPAL measures include MBD, sitting, standing, and steps. ActiGraph measures include SA, LPA, and MVPA.

The orange line provides model adjusted estimates for mean sPPF assuming a linear relationship with the pattern metric, while the blue line provides estimates when using splines to allow for a non-linear relationship. Pointwise 95% confidence intervals are shown with orange and blue shading, respectively. Dotted vertical lines correspond to the 25^th^, 50^th^, and 75^th^ percentiles of the distribution for the given pattern metric. Models include adjustment for awake wear time, age, gender, race/ethnicity, education, body mass index, osteoarthritis, depressive symptoms, Charlson comorbidity index, and MVPA, and incorporate weighting to account for selection into the analytic sample.
